# Immunogenicity of the CYD tetravalent dengue vaccine using an accelerated schedule: randomised phase II study in US adults

**DOI:** 10.1186/s12879-018-3389-x

**Published:** 2018-09-21

**Authors:** Judith Kirstein, William Douglas, Manoj Thakur, Mark Boaz, Thomas Papa, Anna Skipetrova, Eric Plennevaux

**Affiliations:** 1 Advanced Clinical Research, West Jordan, UT USA; 2 Benchmark Research, Sacramento, CA USA; 3 Sanofi Pasteur, Global Clinical Immunology, Discovery Drive, Swiftwater, PA 18370 USA; 4 Sanofi Pasteur, Siège Social, 14 Espace Henry Vallée, 69007 Lyon, France

**Keywords:** Dengue, Live attenuated tetravalent dengue vaccine, Yellow fever, Vaccination schedule

## Abstract

**Background:**

The live attenuated tetravalent dengue vaccine (CYD-TDV) is licensed using a 0-, 6- and 12-month schedule in dengue-endemic areas. An effective shorter schedule may provide more rapid, optimal protection of targeted populations during vaccine campaigns in dengue-endemic countries. We compared immune responses to two schedules of CYD-TDV in a non-endemic population. We also evaluated the impact of yellow fever (YF) co-administration.

**Methods:**

This phase II, open-label, multicentre study enrolled 390 healthy 18–45-year-olds in the USA with no prior exposure to dengue. Participants were randomised (4:4:4:1) to four treatment groups stratified by prior YF vaccine status: Group 1, CYD-TDV standard 0–6–12 months schedule; Group 2, CYD-TDV accelerated 0–2–6 months schedule; Group 3, CYD-TDV accelerated schedule with YF co-administered (dose 1); Group 4, YF vaccination only. Neutralising antibody geometric mean titres (GMTs) and percentages of seropositive participants (antibody titres ≥10 [1/dil]) were measured against each dengue serotype using a 50% plaque reduction neutralisation test.

**Results:**

On D28 post-CYD-TDV dose 3, there were no marked differences in seropositivity rates and GMTs between Groups 1 and 2. In Groups 1 and 2 respectively, 73.4 and 82.4% were dengue seropositive for ≥3 serotypes, with 50.0 and 42.6% seropositive against all four serotypes. Flavivirus status (FV+ or FV−) at baseline did not markedly affect GMTs and seropositivity rates with either schedule. In Groups 1 and 2, GMTs measured 6 months after the third dose decreased against all serotypes, except for a small increase in GMT for serotype 4 in Group 1. In addition, dengue seropositivity remained above 70% for serotypes 2, 3 and 4 in Groups 1 and 2. Co-administration with YF did not affect antibody responses against dengue and YF or impact vaccine safety following completion of the compressed schedule, compared to dengue or YF vaccination alone.

**Conclusions:**

The live attenuated CYD-TDV vaccine given in a compressed schedule in a non-endemic setting can elicit similar antibody responses to the licensed CYD-TDV schedule.

**Trial registration:**

This trial was registered on cinicaltrials.gov, NCT01488890 (December 8, 2011).

**Electronic supplementary material:**

The online version of this article (10.1186/s12879-018-3389-x) contains supplementary material, which is available to authorized users.

## Background

There are an estimated 390 million dengue virus infections worldwide annually, of which around 100 million are associated with clinical manifestations [1, 2]. The majority of infections occur in endemic regions, particularly South East Asia, India, Central and South America, and Africa [1], with dengue also spreading to previously unaffected areas [2].

The live attenuated tetravalent dengue vaccine (CYD-TDV) is approved for use in individuals aged ≥9 years in several endemic countries, administered in 3 doses over a 12-month period (at 0, 6 and 12 months). For endemic populations, an accelerated schedule could provide more rapid protection and increase vaccination compliance in targeted populations. The possibility of using shorter CYD-TDV vaccination schedules therefore needs to be evaluated.

For this study, we elected to describe antibody responses to CYD-TDV in healthy participants in a flavivirus negative, non-endemic setting with the standard vaccination schedule compared to an accelerated, 0–2–6-month schedule. We also evaluated CYD-TDV immunogenicity and safety following concomitant administration with yellow fever (YF) vaccine.

## Methods

### Study design and participants

This phase II, randomised, open-label, multicentre study was conducted between December 2011 and September 2013 in the United States (NCT01488890; December 8, 2011), in accordance with the Declaration of Helsinki and the International Conference on Harmonisation-Good Clinical Practice. The study protocol was approved by an Institutional Review Board which covered each study site. Written informed consent was obtained from all participants. The study adheres to CONSORT reporting guidelines.

Healthy 18–45-year-olds were enrolled. For those reporting previous YF vaccination, documentation (Vaccination Card/record) was provided. Key exclusion criteria were: participation in another clinical trial during the 4 weeks preceding first vaccination; women who were pregnant or of childbearing potential or breastfeeding; receipt of previous flavivirus (FV) vaccine (with the exception of previous YF vaccination for participants classified as YF+).

**Fig. 1:**
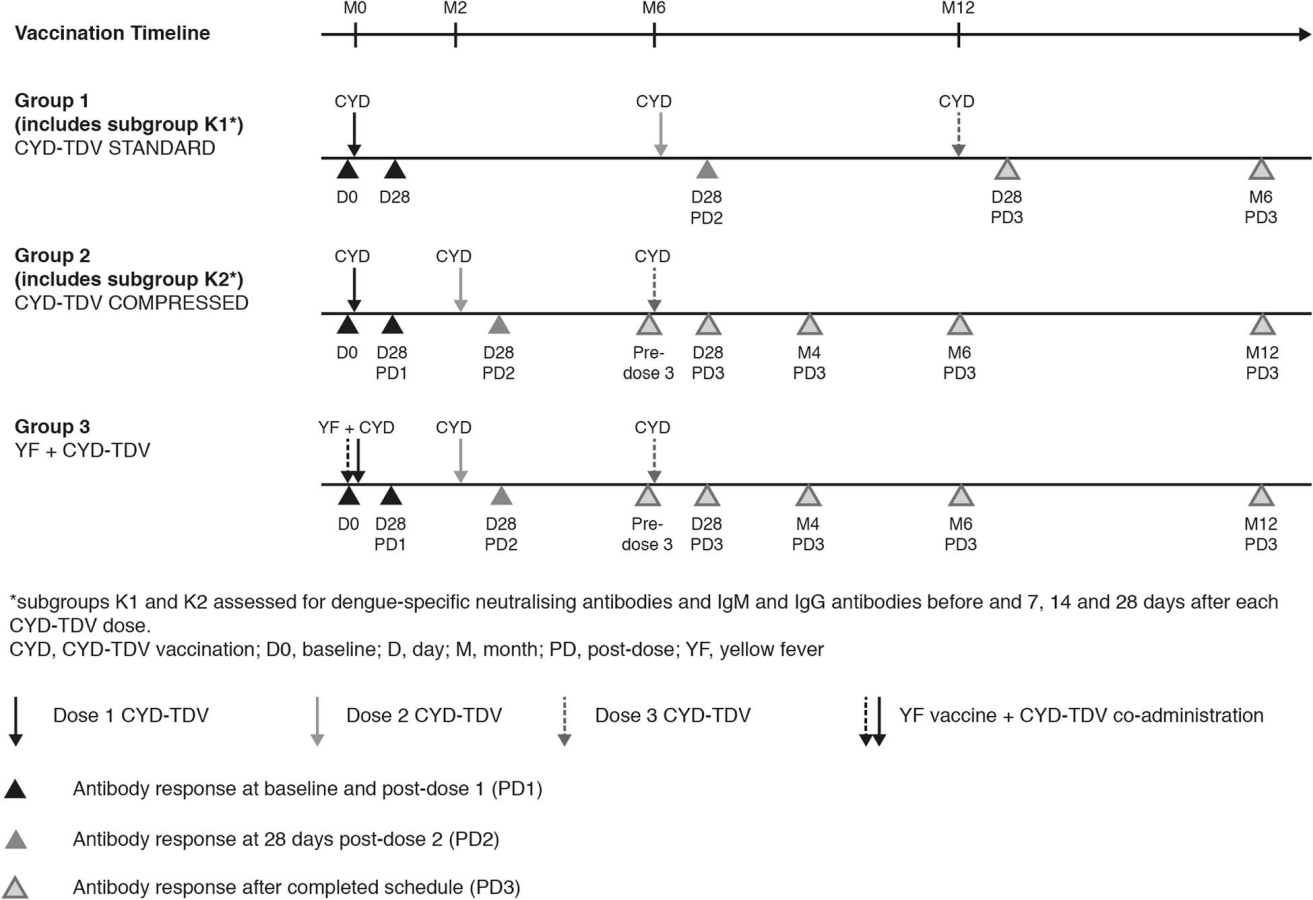
Study design

Participants were randomised (4:4:4:1) to four treatment groups, using the permuted block method with stratification of prior YF vaccine status, to receive CYD-TDV at 0, 6 and 12 months (Group 1; ‘standard’ vaccination schedule); 0, 2 and 6 months (Group 2; ‘compressed’ vaccination schedule); 0, 2 and 6 months with concomitant single YF vaccine dose at month 0 (Group 3); or single YF vaccine dose only (Group 4; not described here). Group 1 and 2 participants were stratified by prior YF vaccination status (YF+ or YF−) in the 3 months to 10 years preceding first study vaccine dose, and randomised such that 50% of Group 1 and 2 participants and no participants in Group 3 had prior reported YF. Equal numbers of YF+ and YF− participants were randomised (*n* = 40 in each) to subsets for antibody kinetic assessment (subgroups K1 and K2). Participants who were YF+ were ineligible for inclusion in Groups 3 or 4. Treatment was allocated using an interactive voice response system or interactive web response. Study duration was 18 months (Fig. 1).

### Vaccines

CYD-TDV was presented as a powder for reconstitution in 0.5 mL saline (NaCl 0.4%) immediately before use. Each 0.5 mL dose contained 5 ± 1 log_10_ cell-culture infectious dose 50% of each of the four live attenuated recombinant CYD-TDV virus serotypes. YF vaccine (YF-VAX^®^; Sanofi-Pasteur) was presented as a powder and reconstituted in saline immediately before use. Each 0.5 mL dose contained ≥4.74 log_10_ plaque forming units of the 17D YF virus strain. Both vaccines were administered subcutaneously into the deltoid region of the upper arm.

### Assessment of CYD-TDV immunogenicity

#### Dengue antibody responses

Dengue neutralising antibody geometric mean titres (GMTs) and seropositivity rates were determined for each dengue serotype at baseline and various intervals following each CYD-TDV dose. Primary endpoints were dengue antibody response at 28 days and 6 months following completion of standard (Group 1) versus compressed (Group 2) vaccination schedules. Secondary immunogenicity endpoints included: dengue antibody responses at 12 months following completion of the compressed schedule in Groups 2 and 3; dengue antibody responses following doses 1 and 2 in Groups 1 and 2 (irrespective of prior YF vaccination); dengue antibody responses by baseline FV seropositivity (defined as seropositivity to dengue or YF, or both) in Groups 1 and 2; and dengue antibody responses in YF− participants from Group 2 versus Group 3 to assess a potential effect of co-administration with YF vaccine.

GMTs were measured for each dengue virus serotype using a dengue 50% plaque reduction neutralisation test (PRNT_50_), performed at Sanofi Pasteur’s Global Clinical Immunology Department (GCI), Swiftwater, Pennsylvania, USA. Baseline YF neutralising antibodies were assessed using PRNT_80_ (Focus Diagnostics Inc., Cypress, California, USA) [3]. Serial two-fold dilutions of serum to be tested, previously heat-inactivated, were mixed with a constant challenge dose of YF vaccinal strain 17D. The mixtures were inoculated in duplicate in wells of a microplate of confluent Vero cells. Following adsorption, cell monolayers were overlaid. The presence of dengue virus infected cells was indicated by plaque formation. Neutralizing antibody titers were calculated and expressed as the reciprocal dilution reducing the mean plaque count by 50%. Negative control wells represented 100% viral load. Dengue or YF seropositivity was defined as antibody titres ≥10 (1/dil), as previously utilized in the vaccine’s clinical development [4] (Additional file 1: Methods).

#### Antibody kinetics

Subgroups K1 and K2 were assessed for dengue-specific neutralising antibodies and IgM and IgG antibodies before and 7, 14 and 28 days after each CYD-TDV dose. IgM and IgG were measured by enzyme-linked immunosorbent assay (ELISA) using commercially available EL1500M and EL1500G DxSelect kits, respectively (Focus Diagnostics Inc., Cypress, California, USA).

#### Blood samples

Blood samples (10 mL) were stored at room temperature for 1–2 h following blood draw and refrigerated; samples were centrifuged within 24 h to collect sera. Sera were stored at or below − 20 °C and shipped frozen for analysis.

### Safety assessment

Participants were monitored for immediate systemic adverse events (AE) during the first 30 min after each vaccine dose. Solicited injection site reactions (pain, erythema, and swelling) were recorded up to 7 days and solicited systemic reactions (fever, headache, malaise, myalgia, and asthenia) up to 14 days after each dose. Unsolicited AEs were recorded up to 28 days. Serious AEs (SAEs) and AEs of special interest (AESIs) were recorded throughout the study.

An early safety review was planned for the first 25% of total participants 14 (+ 2) days after their first vaccination. Enrolment was to be paused if one of the following was observed in CYD-TDV recipients: an unexpected related SAE, ≥20% incidence rate of Grade 3 fever post-dose 1, a serious AESI. No safety concerns were identified at this review, thus enrolment continued as planned.

### Sample size and statistical methods

For Groups 1–3, 120 participants per group gave a probability of 95% for observing at least 1 AE with true incidence of 2.5%, assuming a dropout rate of 15% a total of 102 evaluable participants was anticipated. The sample size was arbitrarily set to 30 participants for Group 4. This was a descriptive study with no formal hypothesis testing. The 95% confidence intervals (CIs) of point estimates were calculated using normal approximation for quantitative data and exact binomial distribution (Clopper-Pearson method) for proportions. It was assumed that neutralising antibody titres were log-normally distributed.

Immunogenicity data were presented for the full analysis dataset, defined as participants who received at least one CYD-TDV or YF vaccine dose and who had at least one blood sample with post-dose serology results. The safety analysis set comprised participants who received at least one CYD-TDV or YF vaccine injection.

## Results

### Participants

**Fig. 2:**
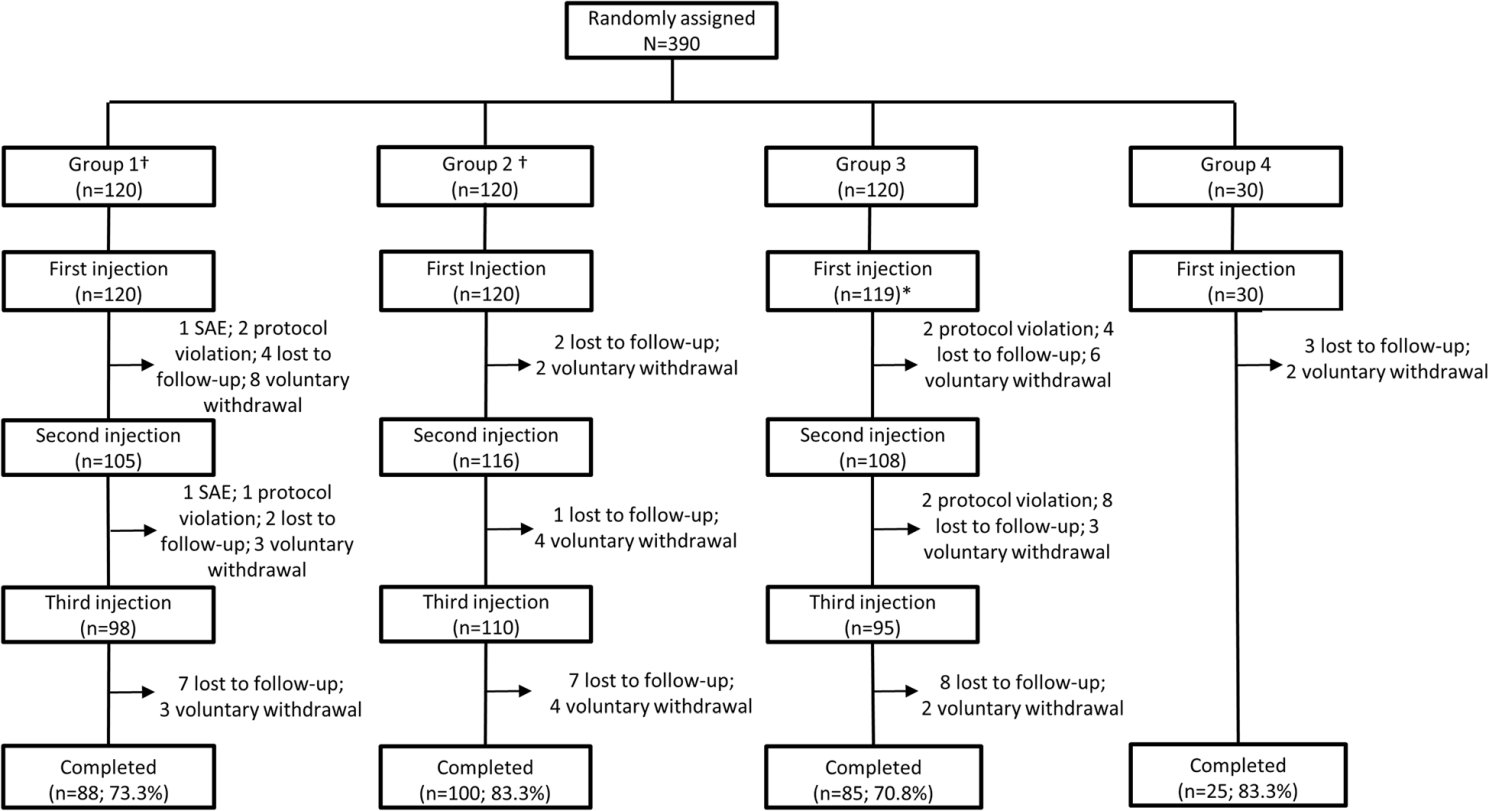
Participant flow through the study (total randomised participants) *Participant presented with Hodgkin’s lymphoma and was discontinued from the study before receiving any vaccine dose †Groups 1 and 2 were further randomised to subgroups K1 (*n* = 40) and K2 (*n* = 40)

A total of 390 participants were included: 120 in each Group 1–3 and 30 in Group 4; 40 participants (20 YF– and 20 YF+) from Groups 1 and 2 were enrolled into subgroups K1 and K2, respectively. Overall, 298 (76.4%) participants completed the study (Fig. 2).

Baseline participant characteristics are summarised in Table 1. Dengue seropositivity at baseline (those with ≥10 [1/dil] for ≥1 serotype) ranged from 7.0 to 7.7%. Of participants who reported prior YF vaccination, 97.5% had YF antibody titres ≥10 (1/dil).

**Table 1:** Participant characteristics at baseline (full analysis set)

	Group 1 (*N* = 117)	Group 2 (*N* = 119)	Group 3 (*N* = 114)
Sex, n (%)			
Female	57 (48.7)	61 (51.3)	58 (50.9)
Age, years			
Mean (SD)	31.8 (7.7)	32.6 (7.3)	32.8 (7.6)
Racial origin			
White Caucasian	88 (75.2)	103 (86.6)	88 (77.2)
Black or African American	21 (17.9)	14 (11.8)	19 (16.7)
Other	8 (6.8)	2 (1.7)	7 (6.1)
Seropositivity status, n (%)^a^			
Dengue seropositive	9 (7.7)	9 (7.6)	8 (7.0)
Yellow fever seropositive^b^	59 (50.4)	61 (51.3)	6 (5.3)
Flavivirus seropositive	63 (53.8)	65 (54.6)	11 (9.6)

*n* number of participants with the specified characteristic, *N* total number of participants in the study group, *SD* standard deviation

^a^Participants were defined as dengue or YF seropositive if they had neutralising antibody titres > 10 1/dilution (for at least one serotype for dengue seropositivity); participants were considered FV seropositive if they were seropositive for dengue of YF, or both

^b^Participants were randomised to treatment groups with stratification on prior reported YF vaccination (in the 3 months to 10 years preceding first study vaccine dose), such that 50% of Group 1 and 2 participants and no participants in Group 3 had prior reported YF. Laboratory confirmation of YF seropositive status according to protocol revealed discrepancies between the YF PRNT_50_ assay and the reported YF vaccination history; the YF seropositive status of participants at baseline was thus re-calculated using YF PRNT_80_, a more stringent assay compared to YF PRNT_50_. Results based on PRNT_80_ are shown for YF and FV seropositive status

### CYD-TDV immunogenicity

#### Impact of compressed CYD-TDV vaccination schedule

Dengue antibody GMTs and percentages of seropositive participants increased for all serotypes following the third CYD-TDV dose of both schedules. There was no marked difference in GMTs by serotype between the two schedules. For both schedules, the highest GMTs were for serotype 4 and the lowest for serotype 1 (Table 2). At 28 days post-dose 3, 73.4% (Group 1) and 82.4% (Group 2) participants were dengue seropositive for ≥3 serotypes, with 50.0 and 42.6% seropositive against all four serotypes.

**Table 2:** Dengue antibody GMTs and seropositivity status in the “standard” dengue vaccination schedule (Group 1) versus the “compressed” dengue vaccination schedule (Group 2)

Time point	Group 1 (*N* = 117)			Group 2 (*N* = 119)		
	n	GMT (95% CI)	Seropositive, % (95% CI)	n	GMT (95% CI)	Seropositive, % (95% CI)
Serotype 1						
Baseline	117	5.38 (4.85–5.96)	1.7 (0.2–6.0)	119	5.13 (4.98–5.28)	2.5 (0.5–7.2)
28 days post-dose 3	93	14.8 (11.3–19.4)	52.7 (42.1–63.1)	108	15.9 (12.6–20.0)	56.5 (46.6–66.0)
6 months post-dose 3	88	13.3 (10.2–17.4)	47.7 (37.0–58.6)	104	9.01 (7.54–10.8)	32.7 (23.8–42.6)
Serotype 2						
Baseline	117	5.19 (4.82–5.58)	0.9 (0.0–4.7)	119	5.22 (4.96–5.50)	2.5 (0.5–7.2)
28 days post-dose 3	94	51.2 (38.2–68.6)	84.0 (75.0–90.8)	108	59.9 (45.8–78.4)	88.0 (80.3–93.4)
6 months post-dose 3	88	45.6 (31.6–65.6)	75.0 (64.6–83.6)	104	38.7 (29.5–50.8)	80.8 (71.9–87.8)
Serotype 3						
Baseline	117	5.32 (4.94–5.73)	2.6 (0.5–7.3)	119	5.28 (5.03–5.55)	4.2 (1.4–9.5)
28 days post-dose 3	94	45.7 (35.0–59.8)	85.1 (76.3–91.6)	107	59.3 (47.0–74.7)	90.7 (83.5–95.4)
6 months post-dose 3	88	30.2 (22.8–40.2)	79.5 (69.6–87.4)	104	34.5 (27.5–43.3)	80.8 (71.9–87.8)
Serotype 4						
Baseline	117	5.78 (5.16–6.48)	6.8 (3.0–13.0)	119	5.11 (4.90–5.33)	0.8 (0.0–4.6)
28 days post-dose 3	94	66.8 (50.9–87.8)	88.3 (80.0–94.0)	107	83.1 (61.4–112.0)	86.0 (77.9–91.9)
6-months post-dose 3	88	74.8 (54.9–102.0)	86.4 (77.4–92.8)	104	41.7 (31.2–55.9)	74.0 (64.5–82.1)
≥ 3 serotypes						
Baseline	117	NA	0.9 (0.0–4.7)	119	NA	0.8 (0.0–4.6)
28 days post-dose 3	94	NA	73.4 (63.3–82.0)	108	NA	82.4 (73.9–89.1)
6-months post-dose 3	88	NA	64.8 (53.9–74.7)	104	NA	58.7 (48.6–68.2)
All 4 serotypes						
Baseline	117	NA	0.9 (0.0–4.7)	119	NA	0.8 (0.0–4.6)
Post-dose 3	94	NA	50.0 (39.5–60.5)	108	NA	42.6 (33.1–52.5)
6-months post-dose 3	88	NA	43.2 (32.7–54.2)	104	NA	26.9 (18.7–36.5)

Seropositive defined as antibody titres ≥10 (1/dil) against each serotype, and against at least 3 or all 4 serotypes with the parental dengue virus strain

*GMT* geometric mean titre, *n* number of participants with available data for endpoint, *NA* not applicable

GMTs, measured 6 months after the third dose, decreased for all serotypes in Groups 1 and 2 with the exception of a small increase in GMT for serotype 4 in Group 1 (Table 2). In addition, dengue seropositivity remained above 70% for serotypes 2, 3 and 4 in Groups 1 and 2.

For the compressed schedule (Groups 2 and 3), no difference in GMTs was observed between 6 and 12 months post-dose 3 for any serotype (Additional file 2: Table S1). Group 1 GMTs were not measured beyond 6 months post-dose 3.

There were no marked differences between compressed and standard schedules in GMTs by serotype 28 days after each of the 3 doses (Additional file 2: Table S2). The 28-day post-dose 2 increase in GMTs was similar for all serotypes irrespective of whether dose 2 was given 2 (Group 2) or 6 months (Group 1) after the first dose (Additional file 2: Table S2). GMTs declined to similar levels pre-dose 3 for both the compressed and standard schedules (Additional file 2: Table S2).

#### Impact of baseline flavivirus status

FV status (FV+ or FV−) did not markedly affect GMTs against serotypes 1, 2 or 3 in Group 1 or for any serotype in Group 2 while serotype 4 GMTs tended to be lower in FV+ than FV− participants in Group 1 (51.7 versus 91.8, 28 days post-dose 3; Table 3). Seropositivity rates were comparable between FV+ and FV− participants in Group 1 for serotypes 1–3 and in Group 2 for serotype 4. However, they tended to be lower in FV+ than FV− participants for serotype 4 in Group 1 (82.7% versus 95.3%) and higher in FV+ participants for serotypes 1–3 in Group 2 (range, 51.0% among FV− to 94.9% among FV+ post-dose 3; Table 3).

**Table 3:** Dengue antibody response post-dose 3 by baseline FV status

	Group 1 (*N* = 117)						Group 2 (*N* = 119)					
	FV Seropositive^a^ (*N* = 63)			FV Seronegative^b^ (*N* = 54)			FV Seropositive^a^ (*N* = 65)			FV Seronegative^b^ (*N* = 54)		
	n	GMT (95% CI)	Seropositive %	n	GMT (95% CI)	Seropositive %	n	GMT (95% CI)	Seropositive %	n	GMT (95% CI)	Seropositive %
Serotype 1												
Baseline	63	5.73 (4.72–6.94)	3.2 (0.4–11.0)	54	5.00 (NC)	0.0 (0.0–6.6)	65	5.23 (4.96–5.53)	4.6 (1.0–12.9)	54	5.00 (NC)	0.0 (0.0–6.6)
28 days post-dose 3	51	15.6 (10.6–23.0)	54.9 (40.3–68.9)	42	13.9 (9.49–20.4)	50.0 (34.2–65.8)	59	17.2 (12.7–23.3)	61.0 (47.4–73.5)	49	14.4 (10.1–20.7)	51.0 (36.3–65.6)
6 months post-dose 3	49	14.8 (10.0–21.7)	53.1 (38.3–67.5)	39	11.7 (8.04–17.1)	41.0 (25.6–57.9)	58	10.3 (7.85–13.4)	36.2 (24.0–49.9)	46	7.63 (6.16–9.46)	28.3 (16.0–43.5)
12 months post-dose 3		NA			NA		55	9.35 (7.50–11.7)	40.0 (27.0–54.1)	44	7.45 (5.87–9.47)	25.0 (13.2–40.3)
Serotype 2												
Baseline	63	5.35 (4.67–6.13)	1.6 (0.0–8.5)	54	5.00 (NC)	0.0 (0.0–6.6)	65	5.42 (4.93–5.96)	4.6 (1.0–12.9)	54	5.00 (NC)	0.0 (0.0–6.6)
28 days post-dose 3	52	60.0 (39.4–91.4)	84.6 (71.9–93.1)	42	42.1 (27.9–63.4)	83.3 (68.6–93.0)	59	68.1 (48.9–94.7)	94.9 (85.9–98.9)	49	51.4 (32.8–80.6)	79.6 (65.7–89.8)
6 months post-dose 3	49	52.5 (32.1–85.8)	79.6 (65.7–89.8)	39	38.1 (21.7–67.0)	69.2 (52.4–83.0)	58	53.0 (37.4–75.2)	91.4 (81.0–97.1)	46	26.0 (17.2–39.3)	67.4 (52.0–80.5)
12 months post-dose 3		NA			NA		56	32.7 (22.6–47.4)	82.1 (69.6–91.1)	44	22.9 (15.5–33.7)	68.2 (52.4–81.4)
Serotype 3												
Baseline	63	5.61 (4.89–6.45)	4.8 (1.0–13.3)	54	5.00 (NC)	0.0 (0.0–6.6)	65	5.53 (5.06–6.05)	7.7 (2.5–17.0)	54	5.00 (NC)	0.0 (0.0–6.6)
28 days post-dose 3	52	44.8 (31.3–64.3)	84.6 (71.9–93.1)	42	46.9 (30.9–71.2)	85.7 (71.5–94.6)	59	71.2 (53.4–94.8)	94.9 (85.9–98.9)	48	47.3 (32.5–69.0)	85.4 (72.2–93.9)
6 months post-dose 3	49	33.0 (23.4–46.5)	85.7 (72.8–94.1)	39	27.1 (16.6–44.3)	71.8 (55.1–85.0)	58	48.5 (36.0–65.3)	87.9 (76.7–95.0)	46	22.5 (16.3–31.0)	71.7 (56.5–84.0)
12 months post-dose 3		NA			NA		56	24.1 (17.6–33.0)	73.2 (59.7–84.2)	44	15.4 (11.1–21.2)	61.4 (45.5–75.6)
Serotype 4												
Baseline	63	6.55 (5.32–8.07)	12.7 (5.6–23.5)	54	5.00 (NC)	0.0 (0.0–6.6)	65	5.20 (4.81–5.63)	1.5 (0.0–8.3)	54	5.00 (NC)	0.0 (0.0–6.6)
28 days post-dose 3	52	51.7 (35.1–76.2)	82.7 (69.7–91.8)	42	91.8 (63.3–133)	95.2 (83.8–99.4)	59	78.5 (54.3–114)	88.1 (77.1–95.1)	48	89.2 (53.3–149)	83.3 (69.8–92.5)
6 months post-dose 3	49	60.0 (38.4–93.8)	81.6 (68.0–91.2)	39	98.7 (64.8–150)	92.3 (79.1–98.4)	58	39.8 (26.9–59.0)	70.7 (57.3–81.9)	46	44.3 (28.3–69.5)	78.3 (63.6–89.1)
12 months post-dose 3		NA			NA		56	46.9 (32.2–68.4)	80.4 (67.6–89.8)	44	48.7 (30.5–77.5)	81.8 (67.3–91.8)

*GMT* geometric mean titre, *n* number of participants with available data for endpoint, *NA* not applicable, *NC* not calculated

^a^FV seropositive participants at baseline defined as: participants with ≥10 1/dil for at least 1 dengue serotype (sera tested by PRNT) or for YF virus (sera with PRNT_80_)

^b^FV seronegative participants are defined as those participants with < 10 1/dil for all dengue serotypes and for YF virus (using sera with PRNT_80_)

#### Impact of YF vaccine co-administration

Dengue antibody response following co-administration with YF versus one dose of CYD-TDV alone is shown in Table 4 for YF− participants. Post CYD-TDV dose 1, GMTs and dengue seropositivity were similar for serotypes 1 and 2 between pooled YF− from Groups 1 and 2 and Group 3 (YF vaccine co-administration), but were higher for serotypes 3 and 4 in Groups 1 and 2 than Group 3 (Table 4).

**Table 4:** Dengue antibody GMTs and seropositivity status, in YF non-immune participants following CYD-TDV alone (Groups 1 and 2 pooled) or CYD-TDV co-administered with YF vaccine (Group 3)

Time point	Group 1 and 2 pooled, YF− (*n* = 116)			Group 3, YF− (*n* = 108)		
	n	GMT (95% CI)	Seropositive, % (95% CI)	n	GMT (95% CI)	Seropositive, % (95% CI)
Serotype 1						
Baseline	116	5.19 (4.87–5.53)	1.7 (0.2–6.1)	108	5.00 (NC)	0 (0.0–3.4)
Post-dose 1	116	8.71 (7.04–10.8)	23.3 (15.9–32.0)	108	7.68 (6.46–9.14)	20.4 (13.2–29.2)
Serotype 2						
Baseline	116	5.06 (4.94–5.19)	0.9 (0.0–4.7)	108	5.06 (4.94–5.20)	0.9 (0.0–5.1)
Post-dose 1	116	18.8 (13.9–25.3)	46.6 (37.2–56.0)	108	10.8 (8.63–13.4)	35.2 (26.2–45.0)
Serotype 3						
Baseline	116	5.21 (4.97–5.47)	2.6 (0.5–7.4)	108	5.28 (4.90–5.69)	2.8 (0.6–7.9)
Post-dose 1	114	28.5 (20.4–39.7)	57.9 (48.3–67.1)	108	8.88 (7.23–10.9)	25.0 (17.2–34.3)
Serotype 4						
Baseline	116	5.43 (4.99–5.92)	3.4 (0.9–8.6)	108	5.26 (4.90–5.65)	1.9 (0.2–6.5)
Post-dose 1	114	229 (139–378)	74.6 (65.6–82.3)	107	22.6 (15.9–32.3)	48.6 (38.8–58.5)

YF seronegative participants at baseline are defined as those participants with YF baseline titre < 10 1/dil using PRNT_80_. Dengue seropositive defined as dengue antibody titres ≥10 (1/dil) against each serotype, and against at least 3 or all 4 serotypes with the parental dengue virus strain

*GMT* geometric mean titre, *n* number of participants with available data for endpoint, *YF* yellow fever

The same trends in GMTs and dengue seropositivity were observed 28 days post-dose 2 (data not shown), but were no longer apparent 28 days post-dose 3. GMTs [95% CI] for Group 2 YF− participants versus Group 3: serotype 3, 47.3 [32.5–69.0] versus 30.2 [22.8–40.1] and serotype 4, 89.2 [53.3–149] versus 71.2 [52.0–97.7]; seropositivity [95% CI]: serotype 3, 85.4% [72.2–93.9] versus 73.5% [62.7–82.6] and serotype 4, 83.3% [69.8–92.5] versus 84.3% [74.7–91.4].

##### Antibody kinetics

For subgroups K1 and K2, dengue IgM levels peaked at 28 days post-dose 1, then declined to the study end (Additional file 2: Figure S1A). The percentage of IgM-positive participants rose from 5.0% pre-dose 1 (both subgroups) to peak values of 72.5% (subgroup K1) and 92.5% (subgroup K2) at 28 days post-dose 1. These percentages dropped to 71.1% at month 2 and 45.7% pre-dose 2 (month 6) for subgroup K1, and 87.5% pre-dose 2 (month 2) for subgroup K2. No substantial increases were observed following doses 2 or 3. Over 50% of participants were IgM-positive up to 14 months post-dose 1 in both subgroups (Additional file 2: Figure S1B).

Dengue IgG levels increased up to 2 months post-dose 1 in both subgroups, and increased further following subsequent doses (Additional file 2: Figure S1A). For both subgroups, the percentage of IgG-positive participants reached at least 70% by 2 months post-dose 1 and 90% by day 14 post-dose 2, remaining above 80% in both subgroups to the study end (Additional file 2: Figure S1B). A limited number of participants (1–2 per subgroup) had a ≥ 4-fold increase in IgG levels post-dose 2 or 3.

##### Safety and reactogenicity of CYD-TDV

The safety profile of CYD-TDV was similar for Groups 1 and 2. No safety concerns were observed following CYD-TDV co-administration with YF (Group 3; Table 5) compared to CYD-TDV alone (Groups 1 and 2) or YF alone (Group 4, Additional file 2: Table S3).

**Table 5:** Safety overview after each CYD-TDV dose – safety analysis set

	Group 1 (*N* = 120)		Group 2 (*N* = 120)		Group 3 (*N* = 119)	
	n/M	% (95% CI)	n/M	% (95% CI)	n/M	% (95% CI)
First CYD-TDV dose						
Solicited reaction	75/118	63.6 (54.2–72.2)	77/119	64.7 (55.4–73.2)	87/115	75.7 (66.8–83.2)
Solicited injection site reaction^a^	34/118	28.8 (20.8–37.9)	42/118	35.3 (26.8–44.6)	40/115	34.8 (26.1–44.2)
Solicited systemic reaction	65/118	55.1 (45.7–64.3)	68/118	57.1 (47.7–66.2)	77/115	67.0 (57.6–75.4)
Unsolicited non-serious AE	34/120	28.3 (20.5–37.3)	32/120	26.7 (19.0–35.5)	27/119	22.7 (15.5–31.3)
Unsolicited non-serious AR	7/120	5.8 (2.4–11.6)	4/120	3.3 (0.9–8.3)	9/119	7.6 (3.5–13.9)
Unsolicited non-serious injection site AR^a^	4/120	3.3 (0.9–8.3)	3/120	2.5 (0.5–7.1)	0/119	0.0 (0.0–3.1)
Unsolicited non-serious systemic AE	31/120	25.8 (18.3–34.6)	30/120	25.0 (17.5–33.7)	25/119	21.0 (14.1–29.4)
Unsolicited non-serious systemic AR	3/120	2.5 (0.5–7.1)	2/120	1.7 (0.2–5.9)	7/119	5.9 (2.4–11.7)
Second CYD-TDV dose						
Solicited reaction	44/101	43.6 (33.7–53.8)	57/114	50.0 (40.5–59.5)	40/96	41.7 (31.7–52.2)
Solicited injection site reaction	21/101	20.8 (13.4–30.0)	32/114	28.1 (20.1–37.3)	24/96	25.0 (16.7–34.9)
Solicited systemic reaction	38/101	37.6 (28.2–47.8)	43/114	37.7 (28.8–47.3)	33/96	34.4 (25.0–44.8)
Unsolicited non-serious AE	15/105	14.3 (8.2–22.5)	18/116	15.5 (9.5–23.4)	15/108	13.9 (8.0–21.9)
Unsolicited non-serious AR	3/105	2.9 (0.6–8.1)	2/116	1.7 (0.2–6.1)	0/108	0.0 (0.0–3.4)
Unsolicited non-serious injection site AR	1/105	1.0 (0.0–5.2)	1/116	0.9 (0.0–4.7)	0/108	0.0 (0.0–3.4)
Unsolicited non-serious systemic AE	15/105	14.3 (8.2–22.5)	18/116	15.5 (9.5–23.4)	15/108	13.9 (8.0–21.9)
Unsolicited non-serious systemic AR	2/105	1.9 (0.2–6.7)	1/116	0.9 (0.0–4.7)	0/108	0.0 (0.0–3.4)
Third CYD-TDV dose						
Solicited reaction	36/91	39.6 (29.5–50.4)	47/107	43.9 (34.3–53.9)	37/89	41.6 (31.2–52.5)
Solicited injection site reaction	19/91	20.9 (13.1–30.7)	29/106	27.4 (19.1–36.9)	21/89	23.6 (15.2–33.8)
Solicited systemic reaction	32/91	35.2 (25.4–45.9)	39/107	36.4 (27.4–46.3)	32/89	36.0 (26.1–46.8)
Unsolicited non-serious AE	19/98	19.4 (12.1–28.6)	17/110	15.5 (9.3–23.6)	13/95	13.7 (7.5–22.3)
Unsolicited non-serious AR	2/98	2.0 (0.2–7.2)	0/110	0.0 (0.0–3.3)	1/95	1.1 (0.0–5.7)
Unsolicited non-serious injection site AR	2/98	2.0 (0.2–7.2)	0/110	0.0 (0.0–3.3)	0/95	0.0 (0.0–3.8)
Unsolicited non-serious systemic AE	17/98	17.3 (10.4–26.3)	17/110	15.5 (9.3–23.6)	13/95	13.7 (7.5–22.3)
Unsolicited non-serious systemic AR	0/98	0.0 (0.0–3.7)	0/110	0.0 (0.0–3.3)	1/95	1.1 (0.0–5.7)

*AE* adverse event, *AR* adverse reaction, *CI* confidence interval, *M* number of participants evaluable for the specified endpoint, *n* number of participants with the specified event

^a^For CYD-TDV injection only; YF safety data not presented here

One participant (Group 3) reported an AE within 30 min after the first CYD-TDV dose (mild [grade 1] dizziness). The percentage of participants reporting solicited reactions was higher following co-administration of CYD-TDV and YF vaccines (Group 3, 75.7%) than following the first CYD-TDV dose (Groups 1 [63.6%] and 2 [64.7%]). In Groups 1–3, solicited reactions were reported less frequently after doses 2 and 3 than after dose 1 and were mostly of grade 1 intensity, started within 3 days after vaccine injection, and resolved spontaneously by day 6. Pain was the most frequently reported injection site reaction after any CYD-TDV dose and was mostly grade 1 intensity, ranging from 28.0% (Group 1) to 34.8% (Group 3) post-dose 1; headache, malaise, and myalgia were the most frequently reported solicited systemic reactions after each dose (29.4% [Group 2, myalgia] to 51.3% [Group 3, headache] post-dose 1).

Unsolicited AEs within 28 days following any injection were reported at similar frequencies for Groups 1, 2, and 3 (13.7–28.3%). Non-serious AEs were mostly systemic, grade 1 or 2 intensity and mostly assessed as not related to vaccination. The percentage of participants reporting an unsolicited adverse reaction (AR) was low across Groups 1–3 (0–7.6%) (Table 5). No AE led to study discontinuation in any group in the 28 days following injection. Three participants reported SAEs within 28 days, none of which were considered related to vaccination. During the trial, 13 participants experienced SAEs: 4 (3.3%) in Groups 1 and 2, and 5 (4.2%) in Group 3. One SAE was reported by the investigator as related to vaccination: a blighted ovum was detected 7 weeks after inadvertent vaccination of a woman during early pregnancy (Group 1; details provided in Additional file 2: Results). Three non-serious AESIs were reported by three participants within 7 days following the first injection; two (flushing of the face [Group 2] and asthma [Group 3]) were assessed as related to vaccination.

## Discussion

This phase II study in healthy adults in a non-endemic area shows that an accelerated 0–2–6-month CYD-TDV vaccination schedule elicits a similar immune response to the licensed 0–6–12-month schedule. More rapid protection would be beneficial in the setting of public health programmes where a shorter schedule may allow potentially better compliance in dengue-endemic populations. Our study also supports that dengue antibody responses to the second CYD-TDV dose can be elicited when given 2 months after the first dose in a non-endemic setting. Notably, two other live attenuated tetravalent dengue vaccines, administered as a single dose [5] or 2 doses 90 days apart [6], are currently in phase III development.

Dengue neutralising antibody GMTs and seropositivity rates after a CYD-TDV vaccination course were lower in our study than in trials undertaken in endemic settings, among adults [7–10] and children [11–13]. Previous large scale studies have demonstrated that a significant proportion of study participants living in endemic areas are FV– prior to vaccination. When dengue antibody responses from FV– participants are compared to the results from this study, the GMTs are very similar: 31.2–75.5 among 2–45-year-olds in Vietnam [10]; 53.4–255 among children in Brazil [14], and 37.6–174 in children in other Latin American countries [12]. Similarly, in adults (18–45 years old) from Singapore, a region of low endemicity, among whom baseline FV seropositivity levels were low, dengue GMTs following vaccination were of a similar magnitude, or only slightly higher depending on serotype, as compared to GMTs observed in the current study [15]. Our findings suggest that the dengue antibody responses elicited following an accelerated vaccine schedule for CYD-TDV are comparable to those elicited by the licensed schedule among adults from non-endemic areas, and appear to be of a similar magnitude to those observed in previous studies among FV-naïve individuals from endemic countries. Further investigation is warranted to determine whether a shorter schedule may be effective in eliciting comparable dengue antibody responses in previously exposed populations from endemic areas.

In our study, a proportion of participants were recruited on the basis of previous YF vaccination; as such, overall baseline FV serostatus can be considered to be driven by baseline YF serostatus. We cannot rule out their exposure to other untested flaviviruses potentially in circulation at the time; the proportion of these previously exposed participants was low and thus unlikely to impact the data. Although some differences in dengue antibody response were observed between FV+ and FV− participants, the differences were inconsistent between serotypes and the number in each sub-group was small, limiting the interpretation of these results. Furthermore, it cannot be excluded that an impact might be observed in a population previously exposed to dengue, YF or another FV. Recent long-term safety data have shown there was an increased risk of hospitalized and severe virologically-confirmed dengue in dengue vaccinated participants who were seronegative; this onset was approximately 3 years after first vaccine dose in 9–16-year-olds [16]. Based on these results, the WHO Strategic Advisory Group of Experts has issued new guidance on the administration of CYD-TDV, with vaccination recommended in seropositive individuals only [17].

As a threshold of protection has not yet been established for dengue using the PRNT assay results, conclusions cannot be drawn on the potential implications of these findings regarding protection against occurrence of symptomatic dengue in these participants. In two pivotal phase III studies, it was suggested that higher post-dose 3 titers were predictive of high vaccine efficacy across all serotypes and age groups [18]. However, protective PRNT_50_ titers may vary by serotype, age and endemicity, as shown in an integrated immunogenicity analysis of CYD-TDV up to 4 years after vaccination [19]. Another limitation of this study is that the PRNT assay does not allow us to differentiate between homotypic antibody responses (believed to confer long term immunity), and heterotypic (cross-reactive) antibody responses (conferring short term cross-protection) [20].

We also observed a lower post-dose 1 dengue antibody response, for serotypes 3 and 4, following co-administration of YF and CYD-TDV vaccines compared to CYD-TDV alone. This potential effect was rectified following the complete vaccination schedule. Further research on the impact of co-administration of FV vaccines is warranted.

The evaluation of IgM antibody kinetics demonstrated that an IgM response may be detected up to several months after vaccination in this study population. IgG levels also increased throughout the vaccination schedule, with > 80% of participants IgG-positive at study end. While these findings are based on a small numbers of participants (40 per group), they suggest that CYD-TDV induces long-lasting dengue neutralising antibody responses and IgM and IgG responses (6–12 months post-dose 1) in this population. This highlights the potential for vaccine-induced false positives in the diagnosis of dengue based on IgM and IgG serological assays, as previously described [21].

The CYD-TDV safety profile in our study was acceptable for both vaccination schedules, and in-line with previous studies [7, 10, 12, 22, 23].

## Conclusions

In conclusion, in a non-endemic setting, CYD-TDV given in a compressed 0–2–6-month schedule can elicit similar antibody responses to the standard 0–6–12-month schedule. Further studies would be needed to evaluate whether this proposed schedule may also apply in endemic populations.

## Additional files

Additional file 1: **Methods.** PRNT assay. Additional information on the methods for the PRNT assay and serostatus at baseline. (DOCX 38 kb)

Additional file 2: **Results.** SAEs during the trial considered related to vaccination**.** Additional information on the SAEs during the trial considered related to vaccination. **Table S1.** Dengue antibody GMTs in the “compressed” dengue vaccination schedule by FV status (Groups 2 and 3) and with YF co-administration (Group 3). **Table S2.** Dengue antibody GMTs in the “standard” dengue vaccination schedule (Group 1) versus the “compressed” dengue vaccination schedule (Group 2) pre-dose and 28 days post-dose during the study. **Table S3.** Safety overview after a single dose of YF vaccine – safety analysis set. **Figure S1.** A. Kinetics of dengue IgG and IgM responses (GMTs [measured by ELISA]; full analysis set). B. Kinetics of dengue IgG and IgM responses (percentage of participants positive for IgM/IgG; full analysis set). (DOCX 392 kb)

## Abbreviations

AE: Adverse event
AESI: Adverse event of special interest
CYD-TDV: Live attenuated tetravalent dengue vaccine
ELISA: Enzyme-linked immunosorbent assay
FV: Flavivirus
GMT: Geometric mean titres
IgG: Immunoglobulin G
IgM: Immunoglobulin M
PRNT: Plaque reduction neutralisation test
SAE: Serious adverse event
YF: Yellow fever
