# Supplementary material for: Immunogenicity of the CYD tetravalent dengue vaccine using an accelerated schedule: randomised phase II study in US adults
Source: BMC Infect Dis. 2018 Sep 21;18:475. doi: 10.1186/s12879-018-3389-x (PMC6150954; doi:10.1186/s12879-018-3389-x)
Supplement: Supplementary file 1 — Methods. PRNT assay. Additional information on the methods for the PRNT assay and serostatus at baseline. (DOCX 38 kb) [file 12879_2018_3389_MOESM1_ESM.docx]

**Additional file 1 – Methods**

**PRNT assay**

In the initial analysis of baseline YF status using PRNT_50_, all participants who had been previously vaccinated against YF had YF antibody titres ≥ 10 1/dil were defined as YF+. However, among participants not previously vaccinated against YF, approximately 30% in each group presented with YF antibody titres ≥ 10 1/dil. Tests were repeated with additional serum aliquots and similar results observed. Given this, samples were re-analysed at 80% plaque reduction (PRNT_80_). In a post-hoc analysis of all groups, among the participants who mentioned that they had been previously vaccinated against YF, 97.5% had YF antibody titres ≥ 10 1/dil, and were thus considered YF immune. Among the participants who reported not to be previously vaccinated against YF, almost all participants in each group (94.8% in Group 1, 96.7% in Group 2, 94.7% in Group 3, and 92.9% in Group 4) presented with YF antibody titres < 10 1/dil, and were thus considered YF non-immune. In both subgroups K1 and K2, 95.0% of the participants who were not previously vaccinated against YF presented with YF antibody titres ≥ 10 1/dil, and were thus considered YF non-immune.

The seropositive or seronegative FV status at baseline was more coherent between the reported information on previous vaccination and the YF PRNT_80_ data, rather than with the PRNT_50_ data. However, the overall conclusions of this additional analysis did not change dramatically between the 2 PRNT analyses. Therefore, results according to YF and FV status at baseline are presented in the manuscript based on the YF PRNT_80_ titre.
