# Supplementary material for: Immunogenicity of the CYD tetravalent dengue vaccine using an accelerated schedule: randomised phase II study in US adults
Source: BMC Infect Dis. 2018 Sep 21;18:475. doi: 10.1186/s12879-018-3389-x (PMC6150954; doi:10.1186/s12879-018-3389-x)

**Additional file 2 – Results**

**SAEs during the trial considered related to vaccination**

A 25-year-old female participant (in Group 1) was inadvertently vaccinated one month after the last menstrual period date, whereas urine pregnancy test was negative on vaccination day. The participant had past obstetric history of four previous pregnancies: two pregnancies terminated by spontaneous abortion, one by elective abortion, and a full term pregnancy with no congenital abnormality. Seven weeks after the vaccination and at approximately nine weeks of pregnancy, blighted ovum was detected at the ultrasound. The event was assessed as related by the Investigator and as not related by the Sponsor. Several months later, during the study, the participant became pregnant again and delivered healthy twins.

**Table S1. Dengue antibody GMTs in the “compressed” dengue vaccination schedule by FV status (Groups 2 and 3) and with YF co-administration (Group 3)**

|  | **Group 2 (N=119)** | | | | **Group 3 (N=114)** | | | |
| --- | --- | --- | --- | --- | --- | --- | --- | --- |
|  | **FV Seropositive ***  **(N=65)** | | **FV Seronegative †**  **(N=54)** | | **FV Seropositive ***  **(N=11)** | | **FV Seronegative †**  **(N=103)** | |
|  | **n** | **GMT (95% CI)** | **n** | **GMT (95% CI)** | **n** | **GMT (95% CI)** | **n** | **GMT (95% CI)** |
| ***Serotype 1*** | | | | | | | | |
| Baseline | 65 | 5.23 (4.96–5.53) | 54 | 5.00 (NC) | 11 | 14.3 (2.79–73.0) | 103 | 5.00 (NC) |
| 6 months post-dose 3 | 58 | 10.3 (7.85–13.4) | 46 | 7.63 (6.16–9.46) | 9 | 21.2 (4.64–96.6) | 79 | 7.38 (6.18–8.81) |
| 12 months post-dose 3 | 55 | 9.35 (7.50–11.7) | 44 | 7.45 (5.87–9.47) | 8 | 43.0 (7.72–239) | 77 | 8.90 (7.05–11.2) |
| ***Serotype 2*** | | | | | | | | |
| Baseline | 65 | 5.42 (4.93–5.96) | 54 | 5.00 (NC) | 11 | 13.5 (3.71–49.4) | 103 | 5.00 (NC) |
| 6 months post-dose 3 | 58 | 53.0 (37.4–75.2) | 46 | 26.0 (17.2–39.3) | 9 | 54.7 (16.8–179) | 79 | 20.2 (14.9–27.5) |
| 12 months post-dose 3 | 56 | 32.7 (22.6–47.4) | 44 | 22.9 (15.5–33.7) | 8 | 58.8 (11.3–306) | 77 | 16.4 (12.1–22.2) |
| ***Serotype 3*** | | | | | | | | |
| Baseline | 65 | 5.53 (5.06–6.05) | 54 | 5.00 (NC) | 11 | 21.5 (6.05–76.2) | 103 | 5.00 (NC) |
| 6 months post-dose 3 | 58 | 48.5 (36.0–65.3) | 46 | 22.5 (16.3–31.0) | 9 | 93.5 (35.1–249) | 79 | 23.0 (16.9–31.1) |
| 12 months post-dose 3 | 56 | 24.1 (17.6–33.0) | 44 | 15.4 (11.1–21.2) | 8 | 113 (50.5–252) | 77 | 13.2 (10.0–17.4) |
| ***Serotype 4*** | | | | | | | | |
| Baseline | 65 | 5.20 (4.81–5.63) | 54 | 5.00 (NC) | 11 | 15.6 (5.33–45.6) | 103 | 5.00 (NC) |
| 6 months post-dose 3 | 58 | 39.8 (26.9–59.0) | 46 | 44.3 (28.3–69.5) | 9 | 64.5 (28.1–148) | 79 | 33.2 (23.4–47.1) |
| 12 months post-dose 3 | 56 | 46.9 (32.2–68.4) | 44 | 48.7 (30.5–77.5) | 8 | 84.2 (27.9–254) | 77 | 33.4 (23.8–46.9) |

CI, confidence interval; FV, flavivirus; GMT, geometric mean titre; n, number of participants with the specified characteristic; N, total number of participants in the study group; NC, not calculated.

* FV seropositive participants at baseline defined as those with ≥ 10 1/dil for at least one serotype with parental dengue virus strain (sera tested by PRNT) or for YF virus (sera with PRNT_80_) result

† FV seronegative participants defined as those with ≥ 10 1/dil for all serotypes with parental dengue virus strains (sera tested by PRNT) and for YF virus (using sera with PRNT_80_ result).

**Table S2. Dengue antibody GMTs in the “standard” dengue vaccination schedule (Group 1) versus the “compressed” dengue vaccination schedule (Group 2) pre-dose and 28 days post-dose during the study**

| **Time point** | **Group 1 (N=117)** | | **Group 2 (N=119)** | |
| --- | --- | --- | --- | --- |
|  | **n** | **GMT**  **(95% CI)** | **n** | **GMT**  **(95% CI)** |
| ***Serotype 1*** | | | | |
| Baseline (pre-dose 1) | 117 | 5.38 (4.85–5.96) | 119 | 5.13 (4.98–5.28) |
| 28 days post-dose 1 | 117 | 10.3 (8.15–13.1) | 118 | 8.75 (7.19–10.6) |
| Pre-dose 2 | 107 | 11.2 (8.76–14.4) | 118 | 11.5 (9.17–14.4) |
| 28 days post-dose 2 | 101 | 18.9 (14.3–25.1) | 116 | 14.9 (11.9–18.6) |
| Pre-dose 3 | 98 | 9.35 (7.47–11.7) | 108 | 9.82 (8.03–12.0) |
| 28 days post-dose 3 | 93 | 14.8 (11.3–19.4) | 108 | 15.9 (12.6–20.0) |
| ***Serotype 2*** | | | | |
| Baseline (pre-dose 1) | 117 | 5.19 (4.82–5.58) | 119 | 5.22 (4.96–5.50) |
| 28 days post-dose 1 | 117 | 30.9 (22.9–41.8) | 119 | 24.2 (18.0–32.4) |
| Pre-dose 2 | 106 | 38.4 (26.2–56.1) | 118 | 40.0 (28.9–55.3) |
| 28 days post-dose 2 | 101 | 64.1 (47.3–86.9) | 116 | 66.9 (50.9–87.9) |
| Pre-dose 3 | 98 | 28.5 (20.3–39.9) | 111 | 46.1 (34.7–61.4) |
| 28 days post-dose 3 | 94 | 51.2 (38.2–68.6) | 108 | 59.9 (45.8–78.4) |
| ***Serotype 3*** | | | | |
| Baseline (pre-dose 1) | 117 | 5.32 (4.94–5.73) | 119 | 5.28 (5.03–5.55) |
| 28 days post-dose 1 | 117 | 32.5 (23.8–44.4) | 117 | 31.0 (22.9–41.8) |
| Pre-dose 2 | 107 | 26.2 (19.3–35.5) | 118 | 34.5 (25.3–47.1) |
| 28 days post-dose 2 | 101 | 47.9 (36.1–63.5) | 116 | 62.0 (46.8–82.2) |
| Pre-dose 3 | 98 | 24.5 (18.8–32.1) | 110 | 37.2 (28.5–48.4) |
| 28 days post-dose 3 | 94 | 45.7 (35.0–59.8) | 107 | 59.3 (47.0–74.7) |
| ***Serotype 4*** | | | | |
| Baseline (pre-dose 1) | 117 | 5.78 (5.16–6.48) | 119 | 5.11 (4.90–5.33) |
| 28 days post-dose 1 | 115 | 121 (74.2–197) | 117 | 126 (77.5–204) |
| Pre-dose 2 | 106 | 60.2 (42.2–85.8) | 117 | 89.8 (58.6–138) |
| 28 days post-dose 2 | 101 | 104 (75.0–144) | 113 | 111 (78.1–159) |
| Pre-dose 3 | 98 | 49.6 (36.2–68.2) | 109 | 74.5 (53.6–104) |
| 28 days post-dose 3 | 94 | 66.8 (50.9–87.8) | 107 | 83.1 (61.4–112) |

CI, confidence interval; GMT, geometric mean titre; n, number of participants with available data for endpoint

| **Table S3. Safety overview after a single dose of YF vaccine – safety analysis set** | | | |
| --- | --- | --- | --- |
|  | **Group 4 (N = 30)** | | |
|  | **n** | **% (95% CI)** | |
| Immediate unsolicited AE | 0 | 0.0 (0.0–11.6) | |
| Immediate unsolicited AR | 0 | 0.0 (0.0–11.6) | |
| Solicited reaction* | 16 | 59.3 (38.8–77.6) | |
| Solicited injection site reaction* | 10 | | 37.0 (19.4–57.6) |
| Solicited systemic reaction* | 16 | | 59.3 (38.8–77.6) |
| Unsolicited AE | 10 | | 33.3 (17.3–52.8) |
| Unsolicited AR | 3 | | 10.0 (2.1–26.5) |
| Unsolicited non-serious AE | 10 | 33.3 (17.3–52.8) | |
| Unsolicited non-serious AR | 3 | 10.0 (2.1–26.5) | |
| Unsolicited non-serious injection site AR | 2 | 6.7 (0.8–22.1) | |
| Unsolicited non-serious systemic AE | 10 | 33.3 (17.3–52.8) | |
| Unsolicited non-serious systemic AR | 1 | 3.3 (0.1–17.2) | |

AE, adverse event; AR, adverse reaction; CI, confidence interval; n, number of participants with specified event

*The number of participants with data available for evaluation of solicited reactions was 27 (30 for all other specified endpoints)

Immediate unsolicited endpoints were evaluated over the 30 minutes following vaccination; solicited AEs were evaluated up to 7 (injection site reactions) or 14 days; unsolicited AEs were evaluated up to 28 days following vaccination.

No deaths, serious AEs, AESIs or AEs leading to study discontinuation were reported following YF vaccination in Group 4.

**Figure S1A. Kinetics of dengue IgG and IgM responses (GMTs [measured by ELISA]; full analysis set)**


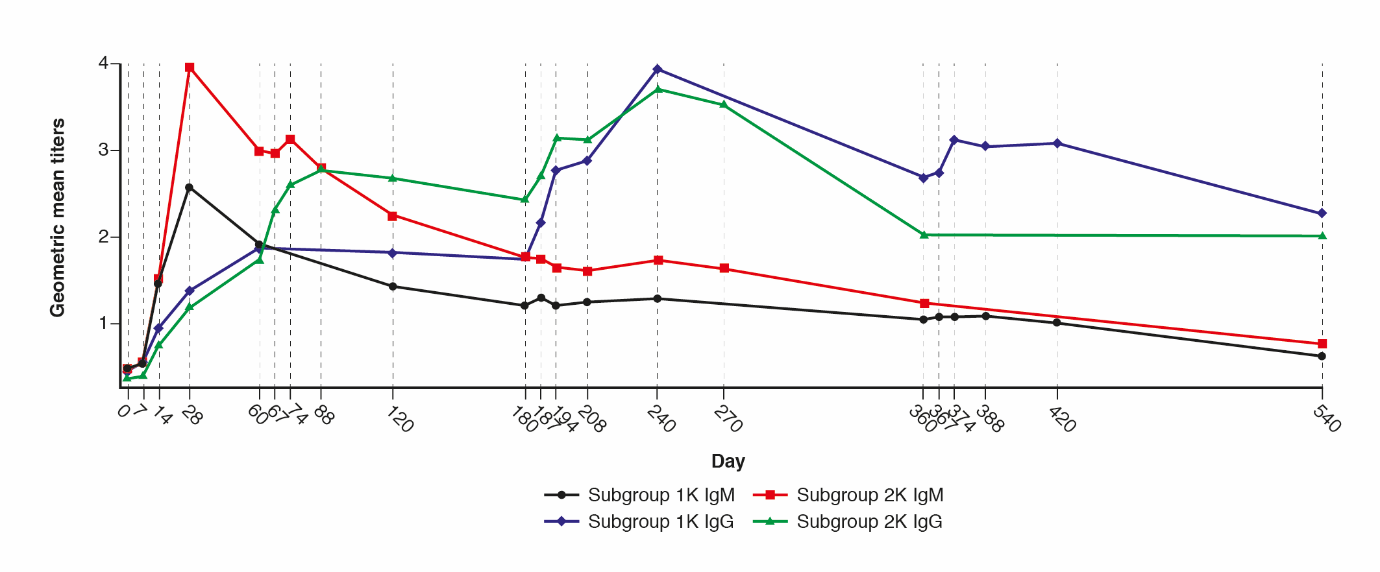


**Figure S1B. Kinetics of dengue IgG and IgM responses (percentage of participants positive for IgM/IgG; full analysis set)**


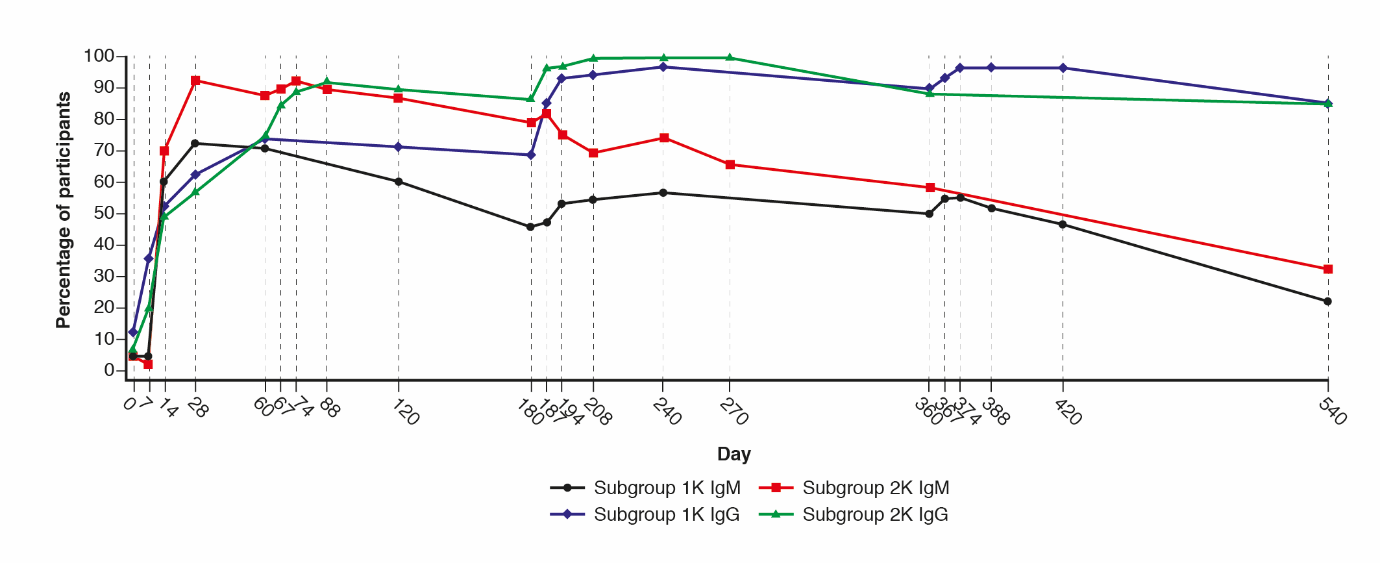

Supplement: Supplementary file 2 — Results. SAEs during the trial considered related to vaccination. Additional information on the SAEs during the trial considered related to vaccination. Table S1. Dengue antibody GMTs in the “compressed” dengue vaccination schedule by FV status (Groups 2 and 3) and with YF co-administration (Group 3). Table S2. Dengue antibody GMTs in the “standard” dengue vaccination schedule (Group 1) versus the “compressed” dengue vaccination schedule (Group 2) pre-dose and 28 days post-dose during the study. Table S3. Safety overview after a single dose of YF vaccine – safety analysis set. Figure S1. A. Kinetics of dengue IgG and IgM responses (GMTs [measured by ELISA]; full analysis set). B. Kinetics of dengue IgG and IgM responses (percentage of participants positive for IgM/IgG; full analysis set). (DOCX 392 kb) [file 12879_2018_3389_MOESM2_ESM.docx]
